# Diverse Bacterial Resistance Genes Detected in Fecal Samples From Clinically Healthy Women and Infants in Australia—A Descriptive Pilot Study

**DOI:** 10.3389/fmicb.2021.596984

**Published:** 2021-09-17

**Authors:** Vanina Guernier-Cambert, Anthony Chamings, Fiona Collier, Soren Alexandersen

**Affiliations:** ^1^Geelong Centre for Emerging Infectious Diseases, Geelong, VIC, Australia; ^2^School of Medicine, Deakin University, Geelong, VIC, Australia; ^3^Barwon Health, University Hospital Geelong, Geelong, VIC, Australia

**Keywords:** AMR, antimicrobial resistance, metagenomics, NGS, resistome, resistance genes

## Abstract

The gut microbiota is an immense reservoir of antimicrobial resistance genes (ARGs), the so-called “resistome.” In Australia, where antibiotic use is high and resistance rates in some common pathogens are increasing, very little is known about the human resistome. To assess the presence and diversity of ARGs in the gut of Australians from south-eastern Victoria, we investigated fecal samples from clinically healthy infants and pregnant women using non-targeted (shotgun metagenomics sequencing or SMS) and targeted sequencing (two Ion Ampliseq^TM^ panels). All methods detected ARGs in all samples, with the detection overall of 64 unique genes conferring resistance to 12 classes of antibiotics. Predominant ARGs belonged to three classes of antibiotics that are the most frequently prescribed in Australia: tetracycline, β-lactams and MLS_B_ (macrolide, lincosamide, streptogramin B). The three bacterial Orders commonly identified as carrying ARGs were Clostridiales, Bacteroidales, and Enterobacteriales. Our preliminary results indicate that ARGs are ubiquitously present and diverse among the gut microbiota of clinically healthy humans from south-eastern Victoria, Australia. The observed resistance pattern partly overlaps with antimicrobial usage in human medicine in Australia, but ARGs to tetracycline are more common than could be expected. Our current sample is small and limited to south-eastern Victoria, and more data on healthy individuals will be needed to better depict resistance patterns at the population level, which could guide population and/or environmental monitoring and surveillance of antibiotic resistance on various spatio-temporal scales in Australia. For future studies, we recommend using the Ion Ampliseq^TM^ Antimicrobial Resistance Research panel, which is sensitive and user-friendly, or combining several methods to increase the detected diversity.

## Introduction

Antimicrobial resistance (AMR) occurs naturally in bacteria and other microorganisms, but in the last decades high exposure to antibiotics has driven the rise and spread of (multi) resistant bacterial pathogens (Roca et al., 2015). According to the World Health Organization, at least 700,000 deaths per year are due to drug resistant diseases, one of the 10 threats to global health in 2019 (World Health Organization, 2018). The concept of an “antibiotic resistome” was first coined to describe the large collection of antimicrobial resistance genes (ARGs) found in a specific environment (D’Costa et al., 2006). Although AMR is most clinically relevant in pathogenic bacteria, ARGs exist in both pathogenic and non-pathogenic bacteria, and large reservoirs of these genes exist in all ecosystems, including all sites of the human or animal body (Aarts and Margolles, 2014) and in natural ecosystems where resistant bacteria can spill over from humans/animals, creating environmental reservoirs for AMR maintenance and spread into new animal hosts (Dolejska and Literak, 2019).

The adult human gastrointestinal tract harbors a vast array of resident bacteria (up to 1,000 phylotypes) which community (the gut microbiota) is shaped by various factors such as age, diet, genetic background, culture, geography, pregnancy, route of delivery, and various chronic conditions and diseases (Fan and Pedersen, 2021). It has been suggested that the human gut resistome is an innate feature of the human gut microbiota which is shaped by other factors such as extensive antibiotic usage (Li et al., 2019; Xia et al., 2019) and class, potency, spectrum and regimen of the antibiotics (Perez-Cobas et al., 2013). While the antibiotic usage is determined by both individual-level consumption and country-level policy, numerous studies have shown that human gut resistomes vary significantly between countries, suggesting that country-specific factors strongly influence the human gut resistome (Xia et al., 2019).

The high rates of community use of systemic antimicrobials in Australia—ranked seventh when compared with 26 European countries and Canada—is considered a serious public health issue, and resistance rates of some key pathogens (e.g., vancomycin-resistant enterococci) are higher in Australia than in Europe (Australian Commission on Safety and Quality in Health Care (ACSQHC), 2019). To date, studies in Australia have focused on ARGs in specific pathogenic bacteria (e.g., *Campylobacter* spp. or *Escherichia coli*), environmental samples (e.g., soils enriched with swine, cattle or poultry manure) (Hu et al., 2016; Zhang et al., 2017; Gou et al., 2018), or water birds (Marcelino et al., 2019), but the gut resistome of the Australian population has not been investigated. We therefore sought to investigate the diversity and abundance of ARGs in a group of clinically healthy infants and pregnant women collected in south-eastern Victoria. While the different methodological approaches to study the gut resistome, as well as their pros and cons, have been reviewed elsewhere (van Schaik, 2015; Clausen et al., 2016), our study was designed as a pilot to evaluate the adequacy of different sequencing methods to achieve an in-depth profile of the resistome specifically in an Australian context.

## Materials and Methods

### Sample Collection

Stool samples were collected from seven adults (HS21-HS26 and HS28) and two infants from southeast Australia. Adult samples were collected from pregnant women at 36 weeks gestation between 2010 and 2013 as part of the Barwon Infant Study (BIS), a birth cohort study with eligibility criteria and cohort profile described elsewhere (Vuillermin et al., 2015). The sampling of infants was opportunistic and unrelated to the BIS study. One infant was sampled at 4 weeks of age (ST5-1mo) and then again at 18 months (ST5-18mo), while the other infant was only sampled at 3 months of age (ST4-3mo). All human stool samples were stored at −80°C prior to DNA extraction.

### Ethics Statement

Ethics approval for the use of the samples from seven pregnant mothers was granted by the Barwon Health Human Research and Ethics Committee (HREC approval 10/24). The study involving the infant samples was deemed negligible or low risk by the Barwon Health Human Research Ethics Committee and therefore exempt from full committee review (HREC approval 17/119).

### Sample Processing and DNA Extraction

DNA was extracted from fecal samples using the Qiagen Powersoil^®^ DNA Isolation Kit (Cat#12888-100) (QIAGEN Pty Ltd., Victoria, Australia) or the QIAamp Fast DNA Stool Mini Kit with Mo Bio bead shearing (Mo Bio, Carlsbad, CA United States) (Supplementary Table 1) with slight modifications to the manufacturer’s protocol following optimization: for the inhibitors’ removal steps, 375 μL of Solution C2 and 335 μL of Solution C3 were used instead of the prescribed volumes. Of note, Qiagen acquired Mo Bio Laboratories in January 2016, with a concomitant change of name of the original Mo Bio extraction kit; so the one kit we used simply had a different name depending on the time of extraction. DNA concentrations were measured using a NanoDrop^TM^ spectrophotometer (Thermo Fisher Scientific, Scoresby, VIC, Australia).

### Library Preparation and Sequencing

Extracted DNA was sequenced using three different Ion Torrent sequencing methods; non-targeted sequencing of sheared DNA, hereafter referred to as “Shotgun Metagenomic Sequencing” (SMS), and targeted metagenomic sequencing by two commercially available Ion Ampliseq^TM^ (PCR) panels (both kindly donated by Life Technologies) designed to target AMR determinants: the Antimicrobial Resistance (AMR) Research Panel and the Pan-Bacterial Research Panel. Reads obtained from the SMS and targeted sequencing were submitted to the European Nucleotide Archive (ENA) under project accession number PRJEB36405.

#### Non-targeted Sequencing: Shotgun Metagenomic Sequencing

Genomic libraries were prepared separately from 100 ng of DNA of each sample. DNA was fragmented using the Ion Shear^TM^ Plus Reagents (Life Technologies, Grand Island, NY) to create a 200 base-read library (15 min enzymatic incubation at 37°C). The adapter ligation and nick repair were performed using the Ion Plus Fragment Library kit and Ion Xpress barcode adapters following the manufacturer’s recommendations (Life Technologies). Sheared DNA was purified with Agencourt AMPure^®^ XP Reagent (1.8× sample volume) (Beckman Coulter, Lane Cove, NSW, Australia). The quantity and size of sheared material was visualized on an Agilent Bioanalyzer DNA 7500 chip (Agilent Technologies) using the High sensitivity DNA kit (Agilent Technologies) assuming a targeted 200 base-read library.

The adapter ligation and nick repair were performed using the Ion Plus Fragment Library kit and IonXpress barcode adapters following the manufacturer’s recommendations (Life Technologies). Ligated and nick repaired DNA was purified with Agencourt AMPure^®^ XP Reagent (1.4× sample volume) (Beckman Coulter) assuming a targeted 200 base-read library. The ligated and nick repaired DNA was size-selected individually with the E-Gel^®^ SizeSelect^TM^ Agarose Gel (Life Technologies). The size selected (unamplified) libraries were quantified as per the manufacturer’s instructions using the Ion Library qPCR Quantitation Kit (Life Technologies) to determine if library amplification was required. When the final library quantity was < 50 pM (i.e., for ST4-3mo) it was further amplified for 10 PCR cycles using Platinum^®^ PCR SuperMix High Fidelity and Library Amplification Primer Mix as per manufacturer’s instructions (Life Technologies) prior to purification and quantification.

All libraries were standardized to a concentration of 100 pM. Libraries were then pooled and diluted to 50 pM prior to loading onto Ion 530^TM^ or 540^TM^ Chips using the Ion Chef Instrument and Ion 530^TM^ or Ion 540^TM^ Kit (Thermo Fisher Scientific). Following template preparation, the chips were run on the Ion Torrent S5xl System (Thermo Fisher Scientific) as per company protocols. Sequencing and associated reactions were performed at the Geelong Centre for Emerging Infectious Diseases (GCEID), Geelong, Victoria, Australia.

#### Targeted Sequencing: IonAmpliSeq^TM^ Panels

We used two commercially available community panels designed to target AMR determinants, both kindly donated by Life Technologies. The first panel was the Ion AmpliSeq^TM^ Pan-Bacterial Research panel, consisting of two primer pools: the one pool contained 269 amplicons targeting 21 specific bacterial species and 716 amplicons targeting 364 known ARGs; the other pool was comprised of 24 amplicons for 16S rRNA gene profiling of up to approximately 400,000 16S rRNA sequences. From this panel, we only used the first primer pool targeting the ARGs.

The second panel was the Ion AmpliSeq^TM^ Antimicrobial Resistance (AMR) Research panel that also consisted of two primer pools comprising a total of 815 amplicons targeting 478 known ARGs (Urbaniak et al., 2018). Both primer pools were used from this panel. The list of primers is available upon request from Life Technologies^1^.

Ten nanograms of extracted DNA were PCR-amplified with the Ion AmpliSeq^TM^ Library Kit (Life Technologies) and Ion AmpliSeq^TM^ Panels, both panels including a 5X Ion AmpliSeq^TM^ HiFi Master Mix. With the Pan-Bacterial Research panel, PCR conditions were: enzyme activation at 99°C for 2 min followed by 18 cycles of 99°C for 15 s and 60°C for 8 min before holding at 10°C. With the AMR Research panel, PCR conditions were: enzyme activation at 99°C for 2 min followed by 19 cycles of 99°C for 15 s and 60°C for 4 min before holding at 10°C. The following steps (adaptors and barcodes ligation, purification, and qPCR quantification) were done as per the manufacturer’s instructions. None of the AmpliSeq^TM^ libraries required further amplification at this stage. Libraries were then pooled prior to loading onto an Ion 530^TM^ Chip using the Ion Chef Instrument. Following template preparation, the chip was run on the Ion Torrent S5xl System (Thermo Fisher Scientific) following company protocols.

### Next Generation Sequence Analyses

**FIGURE 1:**
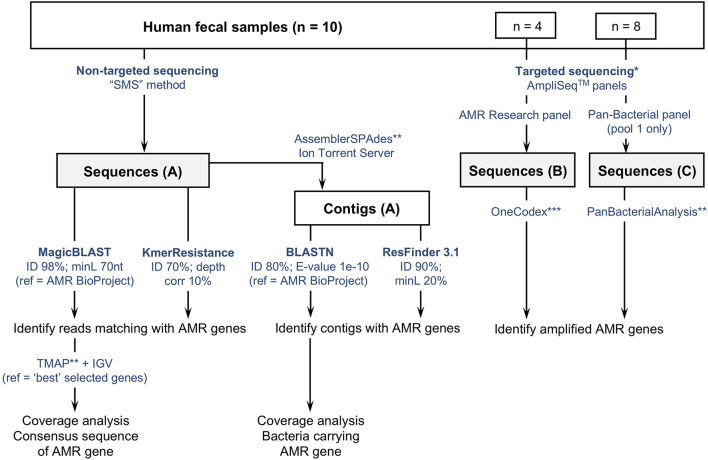
Flowchart describing three culture-independent methods to identify the presence of antimicrobial resistance genes (ARGs) in fecal samples (*n* = 10). **(A)** Non-targeted sequencing of sheared DNA or “Shotgun Metagenomic Sequencing” (SMS) method; **(B,C)** targeted sequencing using two commercially available AmpliSeq^TM^ panels targeting ARGs. Sequences obtained with the SMS method were analyzed using two web-based tools (ResFinder and KmerResistance, https://cge.cbs.dtu.dk/services/), and a method based on the comparison of reads and contigs against the NCBI-curated “Bacterial Antimicrobial Resistance Reference Gene Database” (https://www.ncbi.nlm.nih.gov/bioproject/PRJNA313047). *Targeted sequencing was tested on a subset of samples: 4 with the AMR Research panel, 8 with the Pan-Bacterial Research panel. **refers to Ion Torrent Suite plugins available from the Thermo Fisher Scientific Plugin store. ***Refers to the “One Codex” online platform (available at: https://app.onecodex.com/) used with the AMR Research panel. “ID”: percentage identity threshold; “minL”: minimum length: minimum percentage of coverage compared to reference length or minimum number of nucleotides (nt); “ref”: reference sequences used for comparison with reads and/or contigs.

The three sets of sequence data were analyzed using different tools and methods summarized in Figure 1. Detailed information is provided hereafter and in Supplementary Materials and Methods (especially parameters and settings used with each tool).

#### Analysis of the Shotgun Metagenomic Sequencing Data

The SMS-based data were compared with references in the NCBI-curated “Bacterial Antimicrobial Resistance Reference Gene Database”^2^ containing a total of 4,528 sequences (downloaded on the 2nd of August 2018). Reads were queried against the NCBI database using Magic-BLAST (Boratyn et al., 2019), while contigs were queried using nucleotide BLAST (BLASTn) (Altschul et al., 1990; Mount, 2007).

Individual ARGs coverage and depth metrics were calculated using the Torrent Suite CoverageAnalysis v5.6.0.1 plugin (see Supplementary Materials and Methods for details). The “average base read depth” obtained as an output was used to standardize reads counts; a “mean base depth per 5 million reads” (BD5M) was calculated for each ARG in each sample following the formula: (average base read depth ^∗^ 5,000,000)/total reads. Contrary to the common RPKM (number of reads per kb of gene per million sequenced reads), BD5M is “per base” and not “per read” and its interpretation is independent of the size of the reads or the reference. As an example, if 5 million reads were obtained for a sample, BD5M = 2 for a specific gene is equivalent to reads mapping the whole gene twice or reads mapping half of the gene four times. To avoid confusion between the raw coverage from the sequencer and our calculated BD5M, we hereafter refer to the latest as an abundance rather than a coverage.

In parallel, we also analyzed SMS-based data using two web-based tools—KmerResistance 2.2^3^ (Clausen et al., 2016, 2018) and ResFinder 3.1^4^ (Zankari et al., 2012)—and compared the results with those obtained with our “in-house method” (Figure 1 and Supplementary Materials and Methods).

#### Microbiota Analysis and Host Identification

Binary Alignment Map (BAM) files generated by the Ion Torrent S5xl with the SMS method were uploaded to the Torrent Suite Ion Reporter^TM^ software^5^ in August 2018 for metagenomics analysis of the gut microbiota (see detailed settings in Supplementary Materials and Methods). The relative abundance of the species/genus identified in the gut microbiota was determined from Ion Reporter^TM^. The contigs generated from SMS allowed tentative identification of the bacterial species/orders carrying ARGs. The contigs identified to carry ARGs (when long enough to include some bacterial genome on each side of the resistance gene) were queried against the “nt” and the “WGS” NCBI databases using BLASTn, and against the “Microbes” reference database using BLAST Genomes.

### Statistical Analysis

The *k*-means clustering method was used to investigate the resistance patterns (GENESIS software v.1.8.1, Graz University of Technology, Graz, Austria) (Sturn et al., 2002). Two clusters were selected and the abundance per ARG per sample was provided as an input.

The unpaired *t*-test was used to compare the children group vs. the adult group regarding the total number of ARGs identified, as well as the number of ARGs and total abundance for each class of antibiotics. This statistical analysis was performed using R software v.3.6.1 and a significance level of *P* < 0.05.

## Results

The number of reads and average read lengths generated by each sequencing method are summarized in Supplementary Table 1. Independent of the method used (non-targeted SMS or targeted sequencing with two Ion Ampliseq^TM^ panels), reads that mapped to ARGs were detected in all human samples, with a total of 64 unique ARGs associated with phenotypic resistance to 12 classes of antibiotics. Very technical details and associated problems/pitfalls that we encountered with the different tested methods are provided as Supplementary Results.

### SMS-Based Analysis of ARGs

**FIGURE 2:**
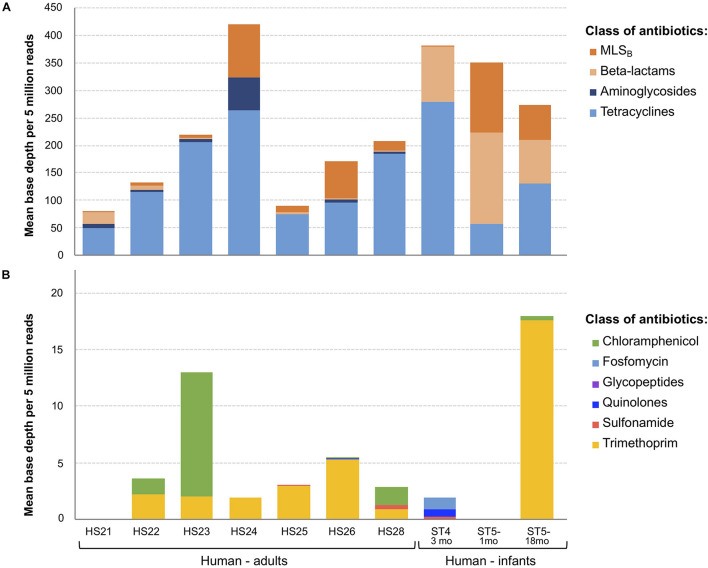
Overview of antimicrobial resistance genes (ARGs) abundance and composition in fecal samples (*n* = 10) using the shotgun metagenomics sequencing (SMS) method. **(A)** Major vs. **(B)** minor ARGs. ARGs abundance was calculated as a “mean base depth per 5 million reads” (BD5M) for each gene in each sample, and then summed for ARGs belonging to the same class of antibiotics.

We identified ARGs from both the reads and the contigs in all 10 samples (Table 1). Detailed results (including the resistance genes identified and the corresponding NCBI references) are provided in Supplementary Tables 2–4. The abundance and composition of the ARGs varied substantially between samples (Table 1 and Figure 2).

**TABLE 1:** Number of antimicrobial resistance genes (ARGs) per class of antibiotics and per sample (*n* = 13).

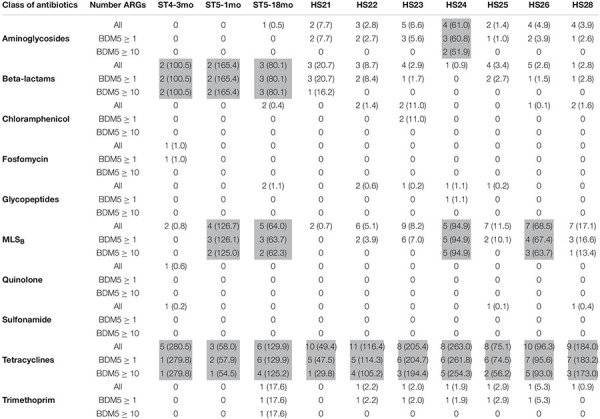

*ARGs were identified from the shotgun metagenomics sequencing (SMS) based on sequence queries against the NCBI “Bacterial Antimicrobial Resistance Reference Gene Database” (BioProject PRJNA313047). Gene abundance was calculated as a mean base depth per 5 million reads (BD5M). In brackets is the total abundance (i.e., the sum of BD5M of all the genes within a group), highlighted in gray when total BD5M ≥ 30 (arbitrary threshold).*

*ST5-1mo, infant 1 month old; ST5-18mo, infant 18 months old; ST4-3mo, infant 3 months old; HS, adult human sample.*

#### Resistance—Antibiotic Classes Level

**FIGURE 3:**
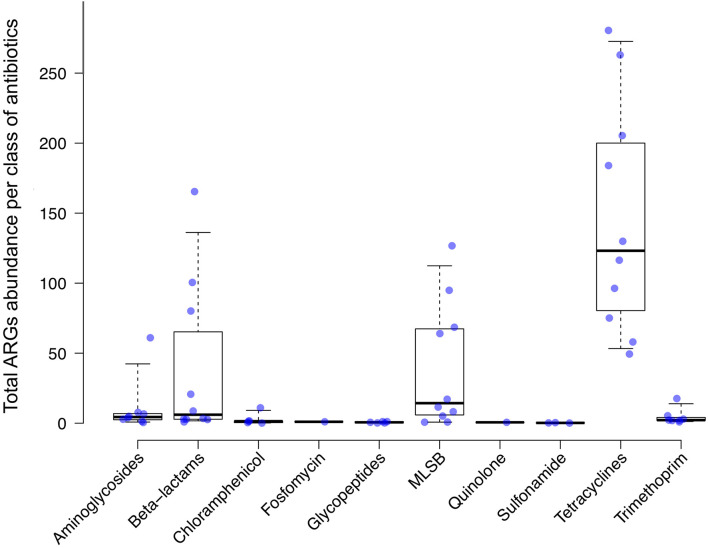
Antimicrobial resistance genes (ARGs) abundance levels. ARGs abundance was calculated as a “mean base depth per 5 million reads” (BD5M) for each gene in each sample, and then summed for ARGs belonging to the same class of antibiotics. Each dot corresponds to a human sample (*n* = 10). Median and 95% confidence intervals are represented.

As BD5M depth coverage could be calculated only for the SMS + BLAST analysis, the results reported in this paragraph refer to this method only. We detected genes conferring resistance to 10 classes of antibiotics (out of 16 classes included in the NCBI’s ARGs database) (Figure 2 and Table 1). ARGs conferring resistance to at least one class of antibiotics at BD5M > 50 were found in all 10 samples. Abundances of genes conferring resistance to the same class of antibiotics were summed for each sample; ARGs conferring resistance to tetracycline (BD5M 49.4–280.5), macrolide, lincosamide, streptogramin B (MLS_B_) (BD5M 0.7–126.7) and β-lactams (BD5M 0.9–165.4) were found in all samples, although at very low abundances in one (for β-lactams) or two samples (for MLS_B_) (Figures 2A, 3 and Table 1). ARGs conferring resistance to tetracycline showed the most variability in abundance between samples, but with an overall pattern of high abundance (median = 123.15; Figure 3). ARGs to aminoglycoside were detected only in adults (BD5M 1.4–61.0). ARGs to six other classes of antibiotics were found at low (trimethoprim and chloramphenicol) to very low abundances (glycopeptides, quinolones, fosfomycin, sulfonamide; BD5M ≤ 1.1) (Figures 2B, 3 and Table 1). The 1-month old infant carried genes conferring resistance to the fewest classes of antibiotics (three classes at BD5M ≥ 1: tetracycline, β-lactams, MLS_B_) while one of the women (HS23) carried ARGs attributable to the most classes of antibiotics (seven classes, or six classes at BD5M ≥ 1) (Table 1).

#### Resistance—Gene Level (ARGs)

At the gene level, we detected 54 unique ARGs from the SMS-generated reads: 47 ARGs using BLAST (or 34 at BD5M ≥ 1) and 7 extra ARGs using the web-based tools (all seven genes were absent from the NCBI’s ARGs database; see Supplementary Figure 1 and Supplementary Table 2 for further comparison of the three methods). For the SMS + BLAST method, that corresponded to an average of 21 ARGs per sample. The lowest diversity of ARGs to one class of antibiotics was observed for fosfomycin, quinolones and trimethoprim (one gene), while the highest diversity was observed for tetracycline (16 genes) and MLS_B_ (13 genes) (Supplementary Table 2).

#### Comparison Between Infants and Adults

At BD5M ≥ 1, the number of ARGs detected in infants (mean = 13.7) was significantly less than in adults (mean = 24.4) (*P* = 0.012); however in most cases the abundance was higher than seen in adults. In infant ST4-3mo for example, two ARGs conferring resistance to β-lactams and one to tetracycline were detected at very high abundances (BD5M ≈ 50 and 280, respectively). When looking at each class of antibiotics independently, we observed significant differences between infants and adults for three classes of antibiotics: aminoglycosides (mean ARGs = 0.33 in infants, mean ARGs = 3.43 in adults; *P* = 0.002), tetracycline (mean ARGs = 4.67 in infants, mean ARGs = 9.14 in adults; *P* = 0.001) and β-lactams (number of ARGs not statistically different, but mean abundance = 115.33 in infants, mean abundance = 44.54 in adults; *P* < 0.001).

**FIGURE 4:**
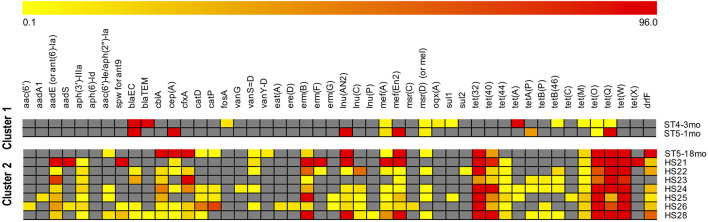
Clustering of samples by antimicrobial resistances genes (ARGs) abundance. ARGs were identified with the SMS + BLAST analysis. The samples identifiers are indicated on the right side (ST are infants, HS are adults). The “mean base depth per 5 million reads” (BD5M) calculated for each ARG in each sample was used as an input for the *k*-means clustering (1,000 maximum iterations, 50 runs). The color scale is: gray when the gene is absent; from light yellow to dark red with increasing gene abundances (BD5M 0.1–96), with values adjusted to log2. To avoid one outlier effect [gene *tet(A)* in ST4-3mo; BD5M = 279.8] the maximum value was set as the second highest abundance [gene *tet(X)* in HS24; BD5M = 96.2].

When comparing the resistance patterns, the *k*-means clustering of ARGs lead to one cluster including the two youngest infants (1mo and 4mo) and the second cluster including all the adults and the oldest infant (18mo) (Figure 4). Samples ST5-1mo and ST5-18mo, despite being collected from the same individual at a different age, did not cluster together.

#### Identification of Bacteria Carrying ARGs

The composition of the fecal microbiota was both individual- and age-related. The results of the metagenomics analysis of the fecal microbiota using the Torrent Suite Ion Reporter^TM^ software are summarized in Table 2, as well as the carrying bacteria of ARGs when identified. The three bacterial Orders commonly identified as carrying ARGs were Clostridiales, Bacteroidales and Enterobacteriales, and the ARGs for which we likely could identify the bacterial host—as identified by a BLASTn query of the contigs carrying these genes—are listed in Table 2. For the rest of the ARGs, the contigs carrying the genes returned poor BLASTn results (no close match in NCBI, or many close matches with references from different bacterial Orders when blasting shorter contigs), preventing the identification of the carrying bacteria. Of note, some ARGs were likely carried by plasmids, e.g., *blaTEM* and *tet(A)* in ST4-3mo, *erm(B)* in HS24.

**TABLE 2:** Percentages of major bacterial Orders in the fecal microbiota of human samples.

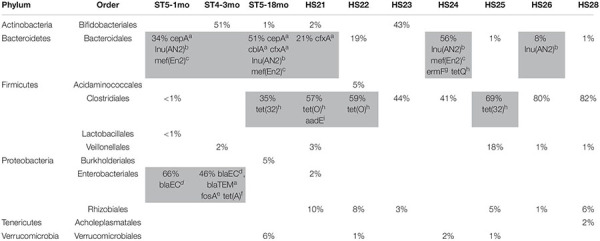

*Bacterial orders likely carrying antimicrobial resistance genes are highlighted in gray and the list of genes is provided.*

*ST5-1mo, infant 1 month old; ST5-18mo, infant 18 months old; ST4-3mo, infant 3 months old; HS, adult human sample.*

*^*a*^Class A broad-spectrum b-lactamase.*

*^*b*^Lincosamide nucleotidyltransferase Lnu(AN2).*

*^*c*^Macrolide efflux MFS transporter.*

*^*d*^Cephalosporin-hydrolyzing class C b-lactamase.*

*^*e*^Fosfomycin resistance glutathione transferase.*

*^*f*^Tetracycline efflux MFS transporter.*

*^*g*^23S rRNA [adenine(2058)-N(6)]-methyltransferase*

*^*h*^Tetracycline resistance ribosomal protection protein.*

*^*i*^Aminoglycoside 6-adenyltransferase.*

### Targeted Sequence Analysis of ARGs

To determine the utility of targeted sequencing, four of the samples were also analyzed using two panels. The results agreed with the ARGs identified by SMS and further detected resistance to two additional classes of antibiotics not detected with the SMS-based methods; Mupirocin and Quaternary Ammonium Compounds. The number of ARGs identified from the four individuals was 29–54 using the AMR panel, 14–30 using the Pan-Bacterial panel, and 9–28 using the SMS-based analysis (Table 3). Even with some primer pairs seemingly poorly efficient (see Supplementary Results), the AMR panel was the most sensitive, with 21–38 ARGs per sample identified exclusively with this method (representing 50.0–66.7% of all ARGs identified in a single individual, all methods combined) (Table 3). This included genes present in the NCBI’s AMR database but nonetheless undetected with the SMS-based method, e.g., *erm(G), sat4, tet(M), lnu(C)*. Despite amplification, those genes were found with the AMR panel at a depth below 6,000 i.e., ≤ 1% of the total amplified reads per sample (see Supplementary Table 5 for details). As a comparison, in sample HS24, all ARGs represented 0.2% of total SMS reads. The Pan-Bacterial panel did not increase much the detected diversity, with only two to three ARGs per sample identified exclusively with this panel (4.4–7.1% of all ARGs) while 5–11 ARGs were identified with the SMS + BLAST analysis alone (11.9–16.2% of all ARGs) (Table 3; see Supplementary Table 5 for a detailed comparison between the SMS + BLAST method and the Ion AmpliSeq^TM^ AMR Research panel).

**TABLE 3:** Number of antimicrobial resistance genes (ARGs) identified with three sequencing methods from four human samples.

	Method	ST5-1mo	HS21	HS24	HS26
Number of ARGs identified with each method	AMR Research panel *	29 (14)	39 (33)	34 (28)	54 (44)
	Pan-Bacterial panel	14	26	23	30
	SMS + BLAST **	9 (7)	17 (10)	20 (16)	28 (15)
Number of unique ARGs identified when overlapping all three methods		34	45	39	64
Number of ARGs identified exclusively with one method ***	AMR Research panel only	24 (66.7%)	27 (55.1%)	21 (50.0%)	38 (55.9%)
	Pan-Bacterial panel only	2 (5.6%)	3 (6.1%)	3 (7.1%)	3 (4.4%)
	SMS + BLAST only	5 (13.9%)	6 (12.2%)	5 (11.9%)	11 (16.2%)

*Reads obtained from the shotgun metagenomics sequencing (SMS) were queried against the NCBI “Bacterial Antimicrobial Resistance Reference Gene Database” (BioProject PRJNA313047) using MagicBLAST. Targeted sequencing used the Ion AmpliSeq^*TM*^ AMR Research panel and the Ion AmpliSeq^*TM*^ Pan-Bacterial Research panel.*

*The number of genes with a depth > 1 is in brackets; depth is defined as the average number of reads piled up across the entire gene.

**The number of genes with a mean base depth per 5 million reads > 1 is in brackets.

***The percentage of ARGs identified with one method only (compared to the total number of ARGs identified with any method) is in brackets.

## Discussion

This study benchmarked the gut resistome of a group of clinically healthy infants and pregnant women from south-eastern Victoria, Australia. We identified diverse antimicrobial resistance genes (at various abundances) that conferred resistance to a broad range of antibiotics and antimicrobials. Our preliminary Australian study confirms results from many other countries that ARGs are ubiquitously present among the gut microbiota of humans, even from a small number of samples.

Using metagenomics on 252 fecal samples collected from residents of Denmark, Spain and the United States, Forslund et al. (2013) identified ARGs to eight classes of antibiotics at an average of 21 ARGs per sample, similar to our SMS results that identified ARGs to 10 classes of antibiotics at an average of 21 ARGs per sample. It has to be noted, however, that gut resistome studies lack international consensus regarding methods and bioinformatics pipelines, leading to very heterogeneous results in the literature (Ho et al., 2020). We greatly increased the diversity of ARGs detected when using targeted sequencing (with the AMR panel) or when combining different methods, with ARGS to two more classes of antibiotics detected. The targeted sequencing methods were very sensitive because of the pre-amplification step by PCR. The AMR Research panel (used, to our knowledge, in a single study before; Urbaniak et al., 2018) identified the highest number of ARGs (up to 54 ARGs in a single individual; at an average of 46 ARGs per sample), i.e., a roughly 100% detection increase of the ARGs diversity.

Overall, the most abundant ARGs identified in clinically healthy Australians conferred resistance to tetracycline, β-lactams and MLS_B_, three classes of antibiotics that are frequently used in human medicine in Australia. It has been suggested that country-specific antibiotic usage impact the human gut resistome, countries with tighter policies on antibiotic usage having considerably less ARGs levels (Xia et al., 2019); therefore, the common usage of specific classes of antibiotics is likely driving the high abundance of matching ARGs in the gut microbiota of people. In our data, ARGs conferring resistance to β-lactams were the most abundant in infants, while ARGs conferring resistance to tetracycline were the most abundant in adults. If we compare this with antimicrobial usage in Australia, β-lactams are the most commonly dispensed antibiotics in human medicine; between 2015 and 2017 in community-based outpatient practice, they accounted for 57.3% of prescriptions, followed by tetracycline (8.0%) and macrolides (5.1%), similar to hospitals prescriptions (63.3% β-lactams; 8.2% tetracycline; 4.0% macrolides) (Australian Commission on Safety and Quality in Health Care (ACSQHC), 2019). Even though this may need to be confirmed with a bigger dataset, the dominance of tetracycline over β-lactams resistance in human adults is consistent with tetracycline resistance being one of the most common types of resistance found in the human gut microbiota worldwide (Hu et al., 2013; Pal et al., 2016). In a study which found that tetracycline resistance genes were the most abundant ARGs in the human gut of all populations from 11 countries (Feng et al., 2018), the authors suggested different factors that could explain this strong dominance, including tetracycline resistance genes could have other important functions not relating to antibiotic resistance, tetracycline resistance is frequently co-selected with other types of resistance as part of multidrug-resistance cassettes, and tetracycline resistance could also be co-selected with food-borne or drug-borne consumption of metals. Those factors could contribute to the observed tetracycline resistance pattern in Australia.

It is difficult to assess how much these preliminary results from a small number of individuals originating from south-eastern Victoria can be transposed at the country level. Interestingly, previous studies have suggested that, while the country of origin has a strong influence on the resistome—linked to the fact that antibiotic usage and exposure is a major driver of the human gut resistome and varies between countries—individual properties such as sex, age, body mass index, or health status have only minor influence on the antibiotic resistance potential of the human gut microbiota (Forslund et al., 2014). This could mean that, even though individual variability is expected, as long as antibiotic usage is similar overall at the country level, human-associated gut resistomes in different geographic regions of Australia might be similar too.

Also, the results from a small-resolution study that analyzed ARGs conferring resistance to tetracycline and macrolides from 20 healthy volunteers from six European countries (Seville et al., 2009) were consistent with later findings on much larger datasets (Forslund et al., 2013, 2014). A bigger sample of Australian volunteers will need to be tested to see if this holds true for our study.

We tested two sequencing techniques and various analysis methods, each having advantages and limitations. The targeted-sequencing methods are limited by their inability to identify novel AMR determinants but they are user-friendly and very sensitive because of the pre-amplification step by PCR (Crofts et al., 2017). Overall, in our data the AMR panel detected the greatest range of ARGs, while the Pan-bacterial Research panel added very little ARGs diversity when compared to the other methods. The SMS-based method was not as sensitive as the targeted methods (being that the sequencing is random with no amplification step) but provided quantitative information (abundance of ARGs) and allowed tentative identification of the host bacteria. Discordant results between methods occurred (i) for ARGs present at low abundances because of different detection sensitivities, and (ii) from the use of different AMR reference databases (e.g., many ARGs identified exclusively with the SMS-based analysis were absent from the AMR panel). Overall, the highest diversity of ARGs was obtained with a combination of two methods: the SMS + BLAST analysis and the AMR panel.

Our data provide information that will help choose a reliable method for the analysis of the resistome in the Australian context. They also provide a first (non-exhaustive) profile of the resistome of clinically healthy women and children in south-eastern Victoria, Australia. The limitation of the study is the small sample size, and a larger study (including more samples and more geographic areas) is needed to better depict resistance patterns at the population level. However, it provides a benchmark for studies to follow, e.g., to explore the differences and similarities between a mother and its child(ren), or the evolution of the resistome with age within a single individual. In addition, further studies are needed to quantify the overlap between the resistome in humans, animals, and the environment in Australia. A national surveillance program of the resistome in the population as well as in pets, farmed or wild animals could be used (i) to follow temporal trends in AMR and detect possible emergences in Australia, and (ii) to inform policy makers and professionals (doctors, veterinarians, farmers) on the most prudent use of critical drugs to ensure we have access to effective treatments of bacterial infections well into the future.

## Data Availability Statement

The datasets presented in this study can be found in online repositories. The names of the repository/repositories and accession number(s) can be found below: https://www.ebi.ac.uk/ena, PRJEB36405.

## Ethics Statement

The studies involving human participants were reviewed and approved by the Barwon Health Human Research and Ethics Committee. Written informed consent to participate in this study was provided by the participants’ legal guardian/next of kin.

## Author Contributions

SA performed the project design and coordination. SA and FC collected and selected the samples, with adult human samples collection coordinated by the BIS investigator group. VG-C and FC performed the DNA extraction, sample library preparation and next generation sequencing. VG-C, AC, and SA performed the reads analyses. VG-C wrote the initial manuscript with input from SA, and later versions were based on input and suggestions from all. All authors contributed to the article and approved the submitted version.

## Conflict of Interest

The authors declare that the research was conducted in the absence of any commercial or financial relationships that could be construed as a potential conflict of interest.

## Publisher’s Note

All claims expressed in this article are solely those of the authors and do not necessarily represent those of their affiliated organizations, or those of the publisher, the editors and the reviewers. Any product that may be evaluated in this article, or claim that may be made by its manufacturer, is not guaranteed or endorsed by the publisher.

## Footnotes

1
https://www.thermofisher.com/

2
https://www.ncbi.nlm.nih.gov/bioproject/PRJNA313047

3
https://cge.cbs.dtu.dk/services/KmerResistance/

4
https://cge.cbs.dtu.dk/services/ResFinder/

5
https://ionreporter.thermofisher.com/ir/

## Supplementary Material

Supplementary Figure 1 Resistance profiles identified from shotgun metagenomics sequencing (SMS) using three analysis methods **(A)** per gene and per sample or **(B)** per gene. SMS-generated reads were screened for the presence of antimicrobial resistance genes using (i) a BLAST comparison based on sequence queries against the NCBI “Bacterial Antimicrobial Resistance Reference Gene Database” (BioProject PRJNA313047), (ii) ResFinder and/or (iii) KmerResistance tools (https://cge.cbs.dtu.dk/services/). BD5M: mean depth coverage per 5 million reads.

Supplementary Table 1 Summary of next generation sequencing results using three methods: (1) shotgun metagenomics sequencing (SMS) or (2) targeted sequencing using community panels from Life Technologies: (2a) the Ion AmpliSeq^TM^ Antimicrobial Resistance (AMR) Research panel and (2b) the Ion AmpliSeq^TM^ Pan-Bacterial Research (PBR) panel. BC: barcode adapter identifier; Reads: number of short read sequences per sample; MRL: mean read length in nucleotides. The four human samples tested with all three methods are in dark gray, the four human samples tested with two methods are in light gray and the five samples tested only with the SMS method are in white.

Supplementary Table 2 Detailed comparison of the analyses with the SMS-based method using (i) the KmerResistance 2.2 tool, (ii) the ResFinder 3.1 tool and (iii) BLAST method. For the SMS + BLAST analysis, the calculated “mean base depth per 5 million reads” is provided (BD5M ≥ 10 is highlighted in red; BD5M ≥ 1 and < 10 is highlighted in yellow; BD5M < 1 is not highlighted). For ResFinder and KmerResistance, the template coverage is provided (as a percentage). ^∗^ Gene absent from the NCBI’s AMR gene database (BioProject PRJNA313047); §gene absent from the ResFinder database.

Supplementary Table 3 Detailed results from the mapping of Shotgun Metagenomic Sequencing (SMS) against the NCBI’s AMR reference gene database using the Torrent Suite TMAP plugin. Reads were queried against the database using Magic-BLAST and contigs were queried against the database using BLASTn. For each reference (line), red squares indicate the samples for which the reference was selected from the reads and/or contigs as the best (or one of the best), while the other samples were mapped against the reference just as a comparison with the other samples. Mean base depth per 5 million reads (BD5M) is highlighted in yellow when ≥ 1 and < 10, or red when ≥ 10. When a reference is the best mapping result but the number of reads mapped to the reference is low (<20), the exact number of reads is provided in brackets.

Supplementary Table 4 Identification of contigs carryings antimicrobial resistance genes using (i) the ResFinder 3.1 tool and (ii) the SMS + BLAST method. Contigs were queried against the NCBI’s AMR reference gene database using either of the two methods. The outputs from ResFinder are provided as available online; column “Alignment length/ref length” originating from the SMS + BLAST method is provided for comparison. For antimicrobial resistance genes detected only by one out of the two methods, the result shows as N/A (not available). ^§^ Gene absent from the ResFinder database; ^∗^ abundance (calculated “mean base depth per 5 million reads” with the shotgun metagenomic sequencing + BLAST analysis) ≤ 1. Blue rows indicate resistance genes identified with the SMS + BLAST method only (i.e., not detected with ResFinder).

Supplementary Table 5 Results from the SMS + BLAST method and a targeted metagenomic sequencing method using the Ion AmpliSeq^TM^ AMR Research panel (Life Technologies). Results obtained with the AMR panel were analyzed with the One Codex online platform (available at: https://app.onecodex.com/); depth is defined as the average number of reads piled up across the entire gene. SMS, shotgun metagenomics sequencing; BD5M, mean base depth per 5 million reads; MLS, macrolide, lincosamide, streptogramin B; QAC, quaternary ammonium compounds; gni, gene not included in Bioproject database; gnp, gene not included in the AMR panel. Genes detected with both methods are highlighted in green; genes in blue are detected with the AMR panel only; genes in yellow are detected with the SMS + BLAST method only.
