# Supplementary material for: Diverse Bacterial Resistance Genes Detected in Fecal Samples From Clinically Healthy Women and Infants in Australia—A Descriptive Pilot Study
Source: Front Microbiol. 2021 Sep 17;12:596984. doi: 10.3389/fmicb.2021.596984 (PMC8484959; doi:10.3389/fmicb.2021.596984)
Supplement: Supplementary file 1 [file Data_Sheet_1.doc]

**Supplementary Material and Methods**

**Details on the next generation sequence analyses**

*Analysis of the sequences obtained by Shotgun Metagenomic Sequencing*

Raw sequences were sorted by barcode/sample using the Ion Torrent Server linked to the Ion Torrent S5xl instrument. Data analysis was based on the comparison of our reads with references in the NCBI-curated “Bacterial Antimicrobial Resistance Reference Gene Database” (<https://www.ncbi.nlm.nih.gov/bioproject/PRJNA313047>) containing a total of 4,528 sequences (downloaded on the 2nd of August 2018). Reads were queried against the NCBI’s ARGs database using Magic-BLAST (Boratyn et al. 2019) with parameters as follows: 98% minimum identity between the read and the reference, and 70 nucleotides minimum length. Concomitantly, the Torrent Suite AssemblerSPAdes 5.6.0 plugin was used to generate contigs from the reads for each sample (Bankevich et al. 2012). These contigs were queried against the NCBI’s ARGs database using nucleotide BLAST (BLASTn) (Altschul et al. 1990, Mount 2007) with parameters as follows: 80% minimum identity between the contig and the reference and 1e-10 minimum E-value.

Due to the scaling factor algorithm used by the Torrent Suite TMAP plugin –designed for mapping reads against a whole genome– issues may arise from mapping against small references like ARGs (1,000 to 2,000 bp). Thus, we artificially increased the length of references used for the mapping by adding chromosomes X, 21 and mitochondrion from the human genome reference sequence (hg19) to each pre-selected ARG sequence (listed in Supplementary Table S1). The extended FASTA references were added to the Ion Torrent Server and used for the mapping of sequence reads using the Torrent Suite TMAP plugin. Mapped reads and consensus sequences were visualised and analysed using the Integrative Genomics Viewer (IGV) (Thorvaldsdottir et al. 2013).

*ARGs coverage and depth*

Individual ARGs coverage and depth metrics were calculated using the Torrent Suite CoverageAnalysis v5.6.0.1 plugin using the filters: library type = whole genome; minimum aligned length = 100; minimum mapping quality = 20. The “average base read depth” obtained as an output was used to standardize reads counts. A “mean base depth per 5 million reads” (or BD5M) was calculated for each ARG in each sample following the formula: (average base read depth * 5,000,000) / total reads. Contrary to the common RPKM (number of reads per kb of gene per million sequenced reads), BD5M is ‘per base’ and not ‘per read’ and its interpretation is independent of the size of the reads or the reference. As an example, if 5 million reads were obtained for a sample, BD5M = 2 for a specific gene is equivalent to reads mapping the whole gene twice or reads mapping half of the gene four times. To avoid confusion between the raw coverage from the sequencer and our calculated BD5M, we hereafter refer to the latest as an abundance rather than a coverage.

*Additional AMR analyses*

We also analysed the sequences obtained with the SMS method using two web-based tools –KmerResistance (available at <https://cge.cbs.dtu.dk/services/KmerResistance/>) (Clausen et al. 2018, Clausen et al. 2016) and ResFinder 3.1 (available at <https://cge.cbs.dtu.dk/services/ResFinder/>) (Zankari et al. 2012)– and compared the results with those obtained with our “in-house method” (Figure 1). Both tools rely on the same reference database (available at <https://cge.cbs.dtu.dk/services/data.php>) (Zankari, Hasman, Cosentino, Vestergaard, Rasmussen, Lund, Aarestrup and Larsen 2012). Raw sequences (FASTQ reads) from each sample were screened for ARGs present in the database with the default settings of 70% identity threshold and 10% depth correction using “bacteria” as the host database and “resistance genes” as the gene database. Scoring method was “species determination on maximum query coverage”. Contigs (FASTA files) from each sample were also screened for ARGs present in the database using the settings of 90% minimum identity and 20% minimum length.

*Microbiota analysis and host identification*

Binary Alignment Map (BAM) files generated by the Ion Torrent S5xl with the SMS method were uploaded to the Torrent Suite Ion Reporter^TM^ software (https://ionreporter.thermofisher.com/ir/) for metagenomics analysis of the gut microbiota. We used a “150-90-3” setting, i.e. minimum read length = 150; minimum alignment coverage between hit and query = 90%; minimum number of unique reads (or ‘read abundance filter’) = 3, meaning that only reads found as triplicates or more were included in the analysis. A minimum percentage identity of 97% was required for genus identification of a ribosomal sequence, and a minimum percentage identity of 99% for species identification. When the top hit and the next best hit differed by less than 0.2%, the sequence was identified as 'species slash ID' (unidentified species). The relative abundance of the species/genus identified in the gut microbiota was determined from Ion Reporter^TM^.

The contigs generated from SMS (when long enough, in order to include some bacterial genome on each side of the resistance gene) allowed tentative identification of host bacteria carrying the ARGs. The contigs identified to carry ARGs were queried against the ‘nt’ and the ‘WGS’ NCBI databases using BLASTn, and against the “Microbes” reference database using BLAST Genomes.

*Analysis of the sequences obtained by Ion AmpliSeq^TM^ panels*

Following targeted amplification, sequences obtained from the Ion Torrent S5xl were analysed based on each sample having a unique barcode and using dedicated platforms or plugins, i.e. (i) the “One Codex” web-based platform (available at: <https://app.onecodex.com/>) for the sequences obtained from using the AMR Research panel, or (ii) the “PanBacterialAnalysis” Torrent Suite plugin from the Ion Torrent Server for the sequences obtained from using the Pan-Bacterial Research panel (see Figure 1). One Codex reported the percentage of the ARG sequence covered by reads in the sample, the percentage identity to the reference ARG sequence, and the depth of read coverage. The “PanBacterialAnalysis” plugin reported the “read counts” matching each ARG in the database.

**References**

Altschul SF, Gish W, Miller W, Myers EW, Lipman DJ. 1990. Basic local alignment search tool. J Mol Biol. Oct 5;215:403-410. Epub 1990/10/05.

Bankevich A, Nurk S, Antipov D, Gurevich AA, Dvorkin M, Kulikov AS, Lesin VM, Nikolenko SI, Pham S, Prjibelski AD, et al. 2012. SPAdes: a new genome assembly algorithm and its applications to single-cell sequencing. J Comput Biol. May;19:455-477. Epub 2012/04/18.

Boratyn GM, Thierry-Mieg J, Thierry-Mieg D, Busby B, Madden TL. 2019. Magic-BLAST, an accurate RNA-seq aligner for long and short reads. BMC Bioinformatics. Jul 25;20:405.

Clausen P, Aarestrup FM, Lund O. 2018. Rapid and precise alignment of raw reads against redundant databases with KMA. BMC Bioinformatics. Aug 29;19:307. Epub 2018/08/31.

Clausen PT, Zankari E, Aarestrup FM, Lund O. 2016. Benchmarking of methods for identification of antimicrobial resistance genes in bacterial whole genome data. J Antimicrob Chemother. Sep;71:2484-2488. Epub 2016/07/02.

Mount DW. 2007. Using the Basic Local Alignment Search Tool (BLAST). CSH Protoc. Jul 1;2007:pdb top17. Epub 2007/01/01.

Thorvaldsdottir H, Robinson JT, Mesirov JP. 2013. Integrative Genomics Viewer (IGV): high-performance genomics data visualization and exploration. Brief Bioinform. Mar;14:178-192. Epub 2012/04/21.

Zankari E, Hasman H, Cosentino S, Vestergaard M, Rasmussen S, Lund O, Aarestrup FM, Larsen MV. 2012. Identification of acquired antimicrobial resistance genes. J Antimicrob Chemother. Nov;67:2640-2644. Epub 2012/07/12.
