# Supplementary material for: Diverse Bacterial Resistance Genes Detected in Fecal Samples From Clinically Healthy Women and Infants in Australia—A Descriptive Pilot Study
Source: Front Microbiol. 2021 Sep 17;12:596984. doi: 10.3389/fmicb.2021.596984 (PMC8484959; doi:10.3389/fmicb.2021.596984)
Supplement: Supplementary file 2 [file Data_Sheet_2.docx]

**Supplementary Results**

We tested two sequencing techniques and analysed the SMS-based data with three methods/tools. The different methods have advantages and limitations. Here we report issues and pitfalls encountered with the different methods that are too detailed/technical to fit in the main manuscript, but are valuable information to avoid misinterpretations of the results.

**Identification of mosaic tetracycline genes**

Mosaic tetracycline resistance genes, presumably arising by recombination between wild-type genes, have been discovered recently, and studies have shown that mosaic genes comprising *tet(O)*, *tet(W)*, and *tet(32)* sequences were abundant in DNA extracted from pig and human fecal samples44. In our data, mosaic genes *tet (O/W), tet(O/32/O)* and *tet(W/32/O)* were identified using the ResFinder tool. There is probably more undetected, as reads mapping against *tet* genes sometimes showed incomplete coverage with clear truncations. Targeted sequencing techniques might be required in order to properly assess tetracycline resistance that might otherwise be underestimated.

**Poor efficiency of some primer pairs in the targeted panels**

Four genes were detected at relatively high abundances with the SMS-based method but at a low depth with the AMR panel: *mef(A), aadE, aadS, tetQ*. This might indicate poor efficiency of some primers. In sample HS24, the “One Codex” online platform reported a mean depth of 328 for *tet(M)* gene when in reality two sets of primers were amplified and detected at different depths (38 and 608). Other sets of primers may be working poorly, leading to false negative results or underestimated depths. Also, genes used to design the primers might also have been being quite different from the one present in our samples. This seemed to be the case for gene *aadE* detected in sample HS24, with the best match using the SMS-based method (NG_047378.1 *Pediococcus acidilactici aadE* gene for aminoglycoside 6-adenylyltransferase) being only 69% similar to the *aadE* gene reference AF516335 used to design the AMR panel.

**Possible false positives with the SMS-based method**

Limitations of the SMS-based in-house analysis include reliance on manual checking of the ARGs detected. In the NCBI database used for the current study, references were constructed to include the ARG but also flanking regions of approximately 100 bp each side (if such regions were available in the original 659 sequence report). This could trigger identification of false-positives for the presence of ARGs. That was the case for *nimJ* gene in HS24 that was originally identified at a BD5M = 0.3, but after manual checking, all reads mapped against the first 100 bp only of reference NG_048017 (*Bacteroides fragilis* *nimJ* gene for nitroimidazole resistance), i.e. the bacterial host genome.

**Possible overestimation of a gene’s abundance**

Manual checking was also essential to avoid overestimation of a gene’s abundance. Gene *tet(C)* was identified in ST4-3mo at a BD5M = 13, but all reads mapped against a limited region of the gene and the consensus sequence was only 80.8% similar to the best reference in the NCBI’s AMR database NG_048174 (*Francisella tularensis tet(C)* gene for tetracycline efflux MFS transporter). The best match overall using BLASTn was gene *tet(A)* (98.9% similarity; reference MG904997). A *tet(A)* gene (reference NG_048158) was actually identified in ST4-3mo at a BD5M = 279.8, confirming that the reads mapped to NG_048174 / *tet(C)* were in fact not the best match.

**Possible underestimation of a gene’s abundance**

In HS24, reference NG_047625 (*Bacteroides fragilis cepA* gene for beta-lactamase) was identified at a BD5M = 0.9 using a Q-value = 20, with NG_047625 being only 80.9% similar to the consensus sequence. This could be verified at a Q-value = 0, with good mapped reads, only quite different from the reference used for mapping; BD5M increased to 4.5, which is probably more accurate. One way to overcome this issue and obtain the real depth is to use the consensus sequence as the reference for re-mapping the reads. Identification from reads or contigs relies heavily on the reference databases, and this example shows that the closest match with a non-perfect reference can still allow the detection of a hypothetical ARG. But because only distantly related to the original curated ARG, further explorations are required to assess if the gene is actually a functional resistance gene.

**Problems associated with library post-amplification**

Library post-amplification (done when the initial library quantity was too low) triggered unexpected problems and should be avoided when possible. Reference NG_047324 (*Escherichia coli aadA1* gene for ant(3”)-Ia family aminoglycoside nucleotidyltransferase) was identified in MAD at a BD5M = 9 but the mapping result showed that all reads matched the same 200 bp region in the middle of the gene, so that the *aadA1* gene coverage was only 22.7%. This specific sample (as well as ST4-3mo, MUD and DFS) was post-amplified, meaning that there might have only been a small amount of *aadA1* gene in the initial DNA extract, and one fragment only was post-amplified and sequenced, leading to overestimating the depth of this gene in MAD.

**Issues associated with AMR databases being non-exhaustive**

All the methods we used rely on reference databases that are non-exhaustive, precluding the detection of ARGs very different to those included in the database, and impacting the abundance estimations of distantly related genes or when multiple similar genes are present. ARGs that show many different alleles were challenging to detect (e.g. *cfxA* / *cfxA6* in sample HS21). For targeted methods, new (larger) panels are constantly being released, e.g. 37,826 probes targeting over 2,000 nucleotide sequences associated with AMR using a probe-and-capture strategy (Guitor, A. K. et al. 2019 Capturing the Resistome: a Targeted Capture Method To Reveal Antibiotic Resistance Determinants in Metagenomes. Antimicrob Agents Chemother 64, doi:10.1128/AAC.01324-19), or 78,600 non-redundant genes (including 47,806 putative ARGs) using targeted metagenomics (Lanza, V. F. et al. 2018 In-depth resistome analysis by targeted metagenomics. Microbiome 6,11, doi:10.1186/s40168-017-0387-y).

Genes for which there is no good match in the reference database or in the NCBI general nucleotide database might be totally missed. When conducting read mapping coverage analyses at a Q-value = 0 instead of 20, we detected in some samples mixed populations of reads, i.e. one population matching the reference quite well, and the second population being more dissimilar. A coverage analysis at a Q-value = 20, showed the real depth of population 1, but discarded population 2 for which no better reference was available.

**Limitations of the online tools for the SMS data analysis**

The ResFinder tool, especially when combined with the KmerResistance tool, had a sensitivity similar to the SMS+BLAST analysis, albeit with the ARGs detected being slightly different. Even though the ResFinder and KmerResistance tools relied on the same database, results did not fully overlap, probably because they used different input data, i.e. contigs versus reads respectively. Compared to the SMS+BLAST analysis, the online tools are more user-friendly and provide quick and relatively accurate results. Provided that the reference database is regularly updated to include missing genes (in our case the obvious ones being aminoglycoside/*aadS*, lincosamide/*lnu(AN2)*, macrolide/*mef(En2)* and b-lactam/*blaEC*) these are valuable tools for scientists and diagnosticians requiring fast results. However, they don’t allow manually rechecking the mapping quality, and do not provide information on whether the genes are chromosomal or on plasmids, nor the taxa of the host bacteria. Even though the ResFinder tool provides an accession number corresponding to a good match for the host bacteria (from the contig sequence), it does provide a single match even though several other (potentially very different) species might show the same percentage of identity with the contig. Also, because we used the ResFinder tool with the contigs only, abundance is not provided, while the KmerResistance tool provides a depth result for the ARGs identified. The SMS-based in-house analysis provided the best accuracy regarding the abundance of ARGs, our calculated “BD5M” coverage taking into account the size of the gene as well as the depth of coverage.
