# Supplementary figures and images for: Diverse Bacterial Resistance Genes Detected in Fecal Samples From Clinically Healthy Women and Infants in Australia—A Descriptive Pilot Study
Source: Front Microbiol. 2021 Sep 17;12:596984. doi: 10.3389/fmicb.2021.596984 (PMC8484959; doi:10.3389/fmicb.2021.596984)

A)

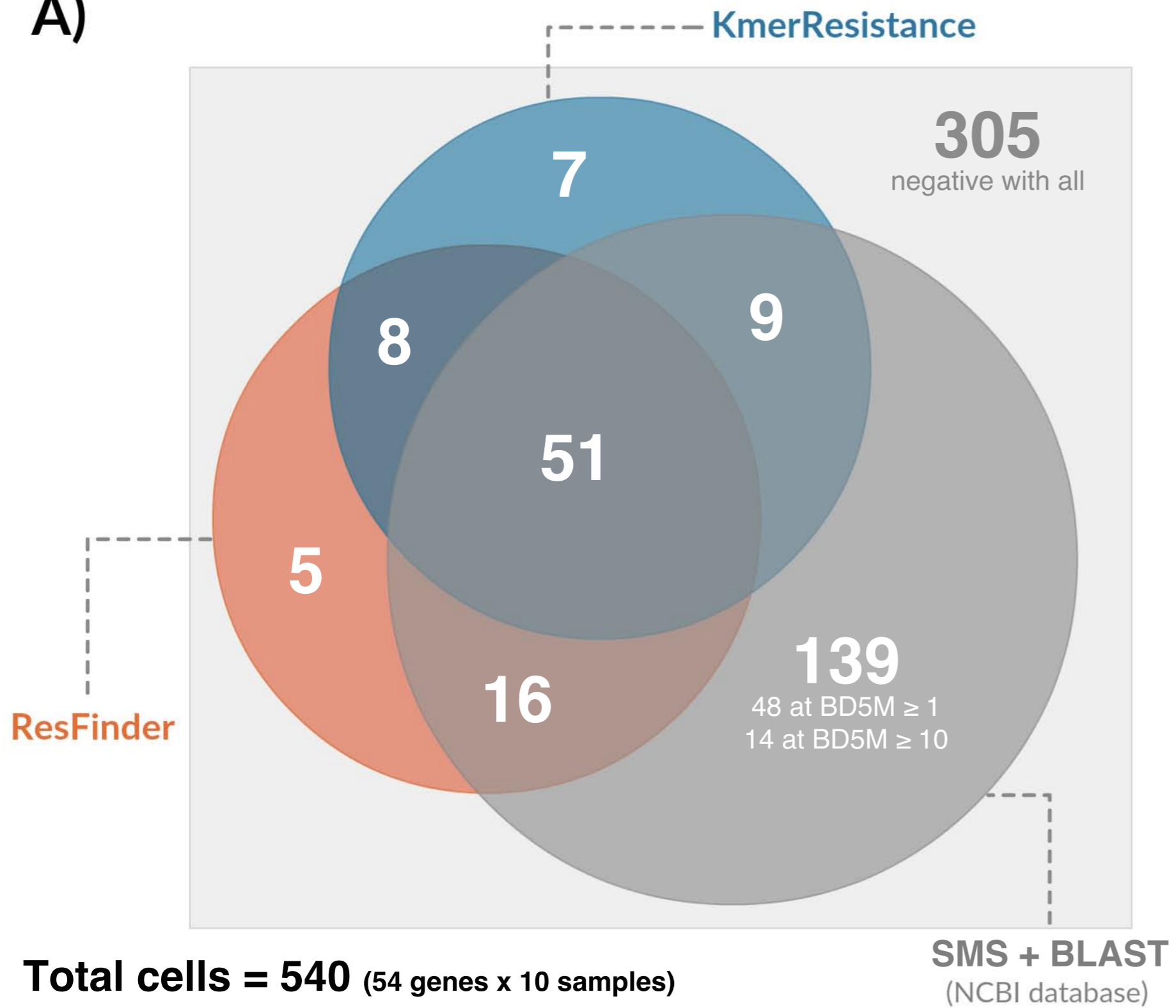

B)

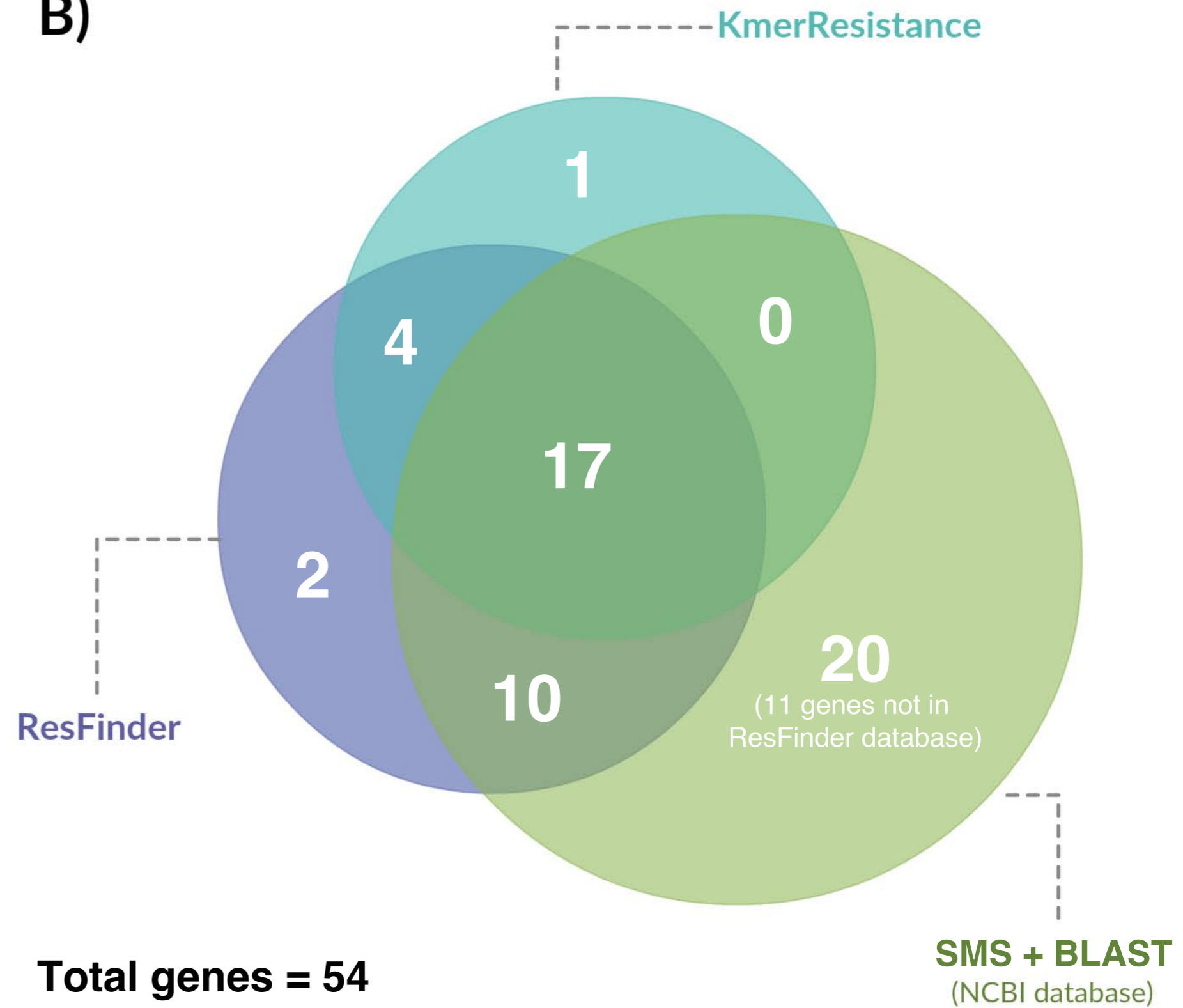

Supplement: Supplementary Figure 1 — Resistance profiles identified from shotgun metagenomics sequencing (SMS) using three analysis methods (A) per gene and per sample or (B) per gene. SMS-generated reads were screened for the presence of antimicrobial resistance genes using (i) a BLAST comparison based on sequence queries against the NCBI “Bacterial Antimicrobial Resistance Reference Gene Database” (BioProject PRJNA313047), (ii) ResFinder and/or (iii) KmerResistance tools (https://cge.cbs.dtu.dk/services/). BD5M: mean depth coverage per 5 million reads. [file Image_1.PDF]
