# Supplementary material for: Diverse Bacterial Resistance Genes Detected in Fecal Samples From Clinically Healthy Women and Infants in Australia—A Descriptive Pilot Study
Source: Front Microbiol. 2021 Sep 17;12:596984. doi: 10.3389/fmicb.2021.596984 (PMC8484959; doi:10.3389/fmicb.2021.596984)
Supplement: Supplementary Table 1 — Summary of next generation sequencing results using three methods: (1) shotgun metagenomics sequencing (SMS) or (2) targeted sequencing using community panels from Life Technologies: (2a) the Ion AmpliSeqTM Antimicrobial Resistance (AMR) Research panel and (2b) the Ion AmpliSeqTM Pan-Bacterial Research (PBR) panel. BC: barcode adapter identifier; Reads: number of short read sequences per sample; MRL: mean read length in nucleotides. The four human samples tested with all three methods are in dark gray, the four human samples tested with two methods are in light gray and the five samples tested only with the SMS method are in white. [file Table_1.doc]

**Supplementary table S1. Extraction methods and NGS sequencing results using three methods:** (1) shotgun metagenomics sequencing (SMS) or (2) targeted sequencing using community panels from Life Technologies: (2a) the Ion AmpliSeq^TM^ Antimicrobial Resistance (AMR) Research panel and (2b) the Ion AmpliSeq^TM^ Pan-Bacterial Research (PBR) panel. BC: barcode adapter identifier; Reads: number of short read sequences per sample; MRL: mean read length in nucleotides. The four human samples tested with all three methods are in dark grey, the four human samples tested with two methods are in light grey and the two samples tested only with the SMS method are in white.

| Sample ID | Host | Extraction method | SMS method | | | AMR panel | | PBR panel | |
| --- | --- | --- | --- | --- | --- | --- | --- | --- | --- |
|  |  |  | BC | Reads | MRL | BC | Reads | BC | Reads |
| ST4-3mo | Human - infant | PowerSoil® DNA Isolation Kit - Mo Bio | BC062 | 5,057,157 | 184 | - | - | - | - |
| ST5-1mo | Human - infant | QIAamp Fast DNA Stool Mini Kit | BC002 | 10,998,563 | 201 | BC049 | 1,486,710 | BC053 | 505,192 |
| ST5-18mo | Human - infant | QIAamp Fast DNA Stool Mini Kit | BC063 | 5,656,858 | 195 | - | - | - | - |
| HS21 | Human - adult | PowerSoil® DNA Isolation Kit - Mo Bio | BC021 | 3,829,581 | 182 | BC050 | 523,118 | BC054 | 524,591 |
| HS22 | Human - adult | PowerSoil® DNA Isolation Kit - Mo Bio | BC022 | 5,721,672 | 184 | - | - | BC055 | 377,819 |
| HS23 | Human - adult | PowerSoil® DNA Isolation Kit - Mo Bio | BC023 | 9,313,335 | 197 | - | - | BC056 | 262,060 |
| HS24 | Human - adult | PowerSoil® DNA Isolation Kit - Mo Bio | BC024 | 7,303,016 | 195 | BC051 | 709,047 | BC057 | 491,234 |
| HS25 | Human - adult | PowerSoil® DNA Isolation Kit - Mo Bio | BC025 | 6,986,354 | 193 | - | - | BC058 | 174,895 |
| HS26 | Human - adult | PowerSoil® DNA Isolation Kit - Mo Bio | BC026 | 9,411,554 | 198 | BC052 | 730,272 | BC059 | 431,487 |
| HS28 | Human - adult | PowerSoil® DNA Isolation Kit - Mo Bio | BC028 | 7,952,415 | 180 | - | - | BC060 | 110,975 |
