# Supplementary material for: Diverse Bacterial Resistance Genes Detected in Fecal Samples From Clinically Healthy Women and Infants in Australia—A Descriptive Pilot Study
Source: Front Microbiol. 2021 Sep 17;12:596984. doi: 10.3389/fmicb.2021.596984 (PMC8484959; doi:10.3389/fmicb.2021.596984)
Supplement: Supplementary Table 4 — Identification of contigs carryings antimicrobial resistance genes using (i) the ResFinder 3.1 tool and (ii) the SMS + BLAST method. Contigs were queried against the NCBI’s AMR reference gene database using either of the two methods. The outputs from ResFinder are provided as available online; column “Alignment length/ref length” originating from the SMS + BLAST method is provided for comparison. For antimicrobial resistance genes detected only by one out of the two methods, the result shows as N/A (not available). § Gene absent from the ResFinder database; ∗ abundance (calculated “mean base depth per 5 million reads” with the shotgun metagenomic sequencing + BLAST analysis) ≤ 1. Blue rows indicate resistance genes identified with the SMS + BLAST method only (i.e., not detected with ResFinder). [file Table_4.docx]

**Supplementary Table S4: Identification of contigs carryings antimicrobial resistance genes using (i) the ResFinder tool and (ii) the SMS + BLAST method.**

SMS: shotgun metagenomic sequencing.

Contigs were queried against the NCBI’s AMR reference gene database using either of the two methods. The outputs from ResFinder are provided as available online; column “Alignment lenght / ref length” originating from the SMS + BLAST method is provided for comparison. For antimicrobial resistance genes detected only by one out of the two methods, the result shows as N/A (not available). Blue rows indicate resistance genes identified with the SMS + BLAST method only (i.e. not detected with ResFinder).

§ gene absent from the ResFinder database ; * abundance ≤ 1 (calculated as “mean base depth per 5 million reads” with the SMS + BLAST analysis).

**Sample ST4-3mo**

| Resistance gene | Identity % | Query/HSP (ResFinder) | Alignment lenght / ref length (BLASTn) | Contig number | Contig length | Position in contig (from ResFinder) |
| --- | --- | --- | --- | --- | --- | --- |
| blaTEM | 100 | 861/861 | 1061/1061 | NODE_171 | 5146 | 247..1107 |
| fosA* | 92.44 | 420/357 | 458/620 | NODE_1762 | 655 | 1..356 |
| mdf(A) | 98.38 | 1233/1233 | N/A | NODE_35 | 90237 | 62780..64011 |
| tet(A) | 100 | 1200/1200 | 1400/1400 | NODE_187 | 3921 | 1548..2747 |
| blaEC § | 98.78 |  | 1234/1234 | NODE_3 | 281368 | N/A |

**Sample ST5-1mo**

| Resistance gene | Identity % | Query/HSP (ResFinder) | Alignment lenght / ref length (BLASTn) | Contig number | Contig length | Position in contig (from ResFinder) |
| --- | --- | --- | --- | --- | --- | --- |
| cep(A) | 100 | 903/903 | 1052/1103 | NODE_35 | 144343 | 68205..69107 |
| mef(A) | 99.83 | 1218/1164 | 1164/1218 | NODE_1496 | 1354 | 193..1354 |
| mdf(A) | 97.89 | 1233/1233 | N/A | NODE_1 | 647648 | 101359..102591 |
| msr(D) (or mel)* | 100 | 1464/516 | 617/1664 | NODE_1859 | 1111 | 1..516 |
| tetA(P) | 99.26 | 1263/1217 | 1294/1463 | NODE_492 | 3338 | 2045..3261 |
| tet(Q) | 100 | 1926/1926 | 2126/2126 | NODE_48 | 110464 | 77139..79064 |
| blaEC § | 99.76 |  | 1234/1234 | NODE_10 | 319723 | N/A |
| lnu(AN2) § | 99.72 |  | 710/713 | NODE_35 | 144343 | N/A |
| mef(En2) § | 99.86 |  | 1406/1406 | NODE_35 | 144343 | N/A |

**Sample ST5-18mo**

| Resistance gene | Identity % | Query/HSP (ResFinder) | Alignment lenght / ref length (BLASTn) | Contig number | Contig length | Position in contig (from ResFinder) |
| --- | --- | --- | --- | --- | --- | --- |
| cepA | 100 | 903/898 | 1103/1103 | NODE_99 | 80984 | 24245..25142 |
| cfxA | 99.79 | 966/966 | 1080/1080 | NODE_64 | 104553 | 103430..104395 |
| tet(32) | 99.90 | 1920/1031 | 1131/2096 | NODE_8240 | 1188 | 159..1188 |
| tet(32) | 99.79 | 1920/943 | 1019/2096 | NODE_2043 | 2871 | 1929..2871 |
| tet(M) | 97.5 | 1920/519 | 550/2032 | NODE_15460 | 766 | 1..518 |
| tet(40) | 99.86 | 1221/705 | 793/1421 | NODE_14779 | 793 | 1..705 |
| tet(Q) | 99.84 | 1926/1926 | 2126/2126 | NODE_199 | 39211 | 20718..22643 |
| tet(W) | 99.90 | 1920/1920 | 2120/2120 | NODE_986 | 5177 | 2694..4613 |
| tet(O/32/O) | 99.53 | 1920/1920 | N/A | NODE_40 | 125390 | 48783..50702 |
| cblA § | 99.91 |  | 1091/1091 | NODE_10 | 264489 | N/A |
| dfrF § | 99.70 |  | 664 | NODE_684 | 7577 | N/A |
| lnu(AN2) § | 99.86 |  | 710/713 | NODE_99 | 80984 | N/A |
| mef(En2) § | 99.79 |  | 1406/1406 | NODE_99 | 80984 | N/A |
| vanY-D* | 98.73 |  | 473/1271 | NODE_30517 | 472 | N/A |

**Sample HS21**

| Resistance gene | Identity % | Query/HSP | Alignment lenght / ref length (BLASTn) | Contig number | Contig length | Position in contig (from ResFinder) |
| --- | --- | --- | --- | --- | --- | --- |
| ant(6)-Ia (aadE) | 100 | 867/583 | 610/994 | NODE_3747 | 2315 | 1..583 |
| ant(6)-Ia (aadE) | 100 | 867/267 | 367/994 | NODE_10675 | 1035 | 769..1035 |
| cfxA6 (cfxA) | 99.6 | 996/996 | 947/1166 | NODE_8811 | 1242 | 124..1119 |
| tet(W) | 98.91 | 1920/548 | 548/2120 | NODE_20633 | 616 | 1..548 |
| tet(W/32/O) | 99.19 | 1920/740 | 843/2120 | NODE_11960 | 935 | 1..740 |
| tet(O/W) | 98.64 | 1889/664 | 664/2120 | NODE_17699 | 686 | 23..686 |
| tet(Q) | 99.84 | 1926/1926 | 2126/2126 | NODE_609 | 7704 | 3887..5812 |
| blaEC § | 98.92 |  | 278/1234 | NODE_21234 | 604 | N/A |
| tet(32) | 81.08 |  | 1152/2096 | NODE_9485 | 1151 | N/A |
| tet(O) | 99.30 |  | 428/2120 | NODE_8966 | 1120 | N/A |

**Sample HS22**

| Resistance gene | Identity % | Query/HSP | Alignment lenght / ref length (BLASTn) | Contig number | Contig length | Position in contig (from ResFinder) |
| --- | --- | --- | --- | --- | --- | --- |
| erm(B) | 99.57 | 747/470 | 470/938 | NODE_90943 | 469 | 1..469 |
| catS | 99.55 | 492/220 | 300/829 | NODE_90619 | 470 | 251..470 |
| tet(W) | 100 | 1920/1470 | 1570/2120 | NODE_3333 | 3312 | 1843..3312 |
| tet(W) | 96.40 | 1920/444 | 444/2120 | NODE_99286 | 444 | 1..444 |
| tet(W) | 95.92 | 1920/490 | 490/1920 | NODE_85022 | 490 | 1..490 |
| tet(W) | 96.36 | 1920/439 | 447/2120 | NODE_98165 | 447 | 9..447 |
| tet(Q) | 99.88 | 1926/1601 | 1607/2126 | NODE_11503 | 1696 | 1..1601 |
| tet(Q) | 99.30 | 1926/426 | 526/2126 | NODE_1219 | 5353 | 4928..5353 |
| tet(32) | 99.88 | 1920/864 | 978/2096 | NODE_23583 | 1106 | 230..1093 |
| tet(32) | 99.86 | 1920/726 | 802/2096 | NODE_13661 | 1532 | 808..1532 |
| tet(40) | 99.75 | 1221/1221 | 1339/1421 | NODE_3333 | 3312 | 354..1574 |
| tet(W/32/O) | 99.67 | 1920/903 |  | NODE_12013 | 1657 | 756..1657 |
| tet(O/W) | 93.97 | 1889/448 |  | NODE_98041 | 447 | 1..447 |
| tet(O/W) | 94.73 | 1889/664 |  | NODE_40594 | 783 | 1..664 |
| tet(O) | 99.64 | 1920/1920 | 2120/2120 | NODE_3146 | 3401 | 533..2451 |
| cblA § | 99.52 |  | 628/1091 | NODE_57524 | 628 | N/A |
| catD § * | 84.67 |  | 300/829 | NODE_90619 | 470 | N/A |
| catP* | 84.67 |  | 300/824 | NODE_90619 | 470 | N/A |

**Sample HS23**

| Resistance gene | Identity % | Query/HSP | Alignment lenght / ref length (BLASTn) | Contig number | Contig length | Position in contig (from ResFinder) |
| --- | --- | --- | --- | --- | --- | --- |
| ant(6)-Ia (aadE) | 99.77 | 867/867 | 994/994 | NODE_3228 | 3819 | 1826..2691 |
| ant(6)-Ii (aac(6’))* | 97.81 | 549/320 | 420/749 | NODE_41932 | 603 | 284..603 |
| cfxA | 99.75 | 966/785 | 837/1034 | NODE_4155 | 2999 | 2215..2999 |
| msr(C) | 96.85 | 1479/1016 | 1021/1479 | NODE_15648 | 1114 | 101..1114 |
| ere(D) | 93.80 | 1227/693 | 755/1389 | NODE_21835 | 903 | 212..903 |
| erm(B) | 98.24 | 738/738 | 841/938 | NODE_24364 | 840 | 93..829 |
| catS | 98.82 | 492/422 | 500/829 | NODE_57486 | 499 | 78..499 |
| tet(40) | 99.77 | 1221/856 | 956/1421 | NODE_5731 | 2260 | 1405..2260 |
| tet(40) | 99.73 | 1221/372 | 390/1421 | NODE_51101 | 536 | 1..372 |
| tet(W) | 100 | 1920/427 | 527/2120 | NODE_1544 | 7966 | 1..427 |
| tet(W) | 100 | 1920/427 | 527/2120 | NODE_4157 | 2999 | 1..427 |
| tet(W) | 99.37 | 1920/1592 | 1611/2120 | NODE_8873 | 1611 | 20..1611 |
| tet(Q) | 96.12 | 1926/516 | 516/2126 | NODE_54378 | 516 | 1..516 |
| tet(Q) | 99.84 | 1926/1926 | 2126/2126 | NODE_1478 | 8301 | 5002..6927 |
| tet(O/W) | 92.69 | 1889/547 |  | NODE_47216 | 561 | 1..547 |
| tet(32) | 99.85 | 1920/684 | 760/2096 | NODE_13882 | 1201 | 1..683 |
| tet(32) | 99.82 | 1920/1106 | 1206/2096 | NODE_11608 | 1344 | 240..1344 |
| tet(O) | 99.74 | 1920/1920 | 2120/2120 | NODE_4010 | 3105 | 386..2305 |
| aadS § * | 99.61 |  | 763/1064 | NODE_4392 | 2839 | N/A |
| catD § | 84.29 |  | 643/829 | NODE_2867 | 4316 | N/A |
| catP | 84.76 |  | 656/824 | NODE_2867 | 4316 | N/A |
| dfrF § | 100 |  | 658/695 | NODE_9203 | 1572 | N/A |
| eat(A) § * | 100 |  | 338/1703 | NODE_14705 | 1157 | N/A |
| lnu(AN2) § | 100 |  | 222/713 | NODE_28931 | 756 | N/A |
| mef(En2) § | 99.72 |  | 710/1406 | NODE_28931 | 756 | N/A |

**Sample HS24**

| Resistance gene | Identity % | Query/HSP | Alignment lenght / ref length (BLASTn) | Contig number | Contig length | Position in contig (from ResFinder) |
| --- | --- | --- | --- | --- | --- | --- |
| ant(6)-Ia (aadE) | 99.88 | 867/867 | 994/994 | NODE_2869 | 5385 | 4425..5291 |
| erm(F) | 99.63 | 801/801 | 969/1001 | NODE_18891 | 1268 | 164..964 |
| mef(A) | 95.07 | 1218/1218 | 667/1010 | NODE_44 | 63779 | 305..1522 |
| erm(B) | 99.59 | 738/738 | 889/938 | NODE_555 | 17398 | 7320..8056 |
| tet(Q) | 90.26 | 1926/616 | 623/2126 | NODE_48191 | 625 | 10..625 |
| tet(Q) | 97.41 | 1926/1702 | 1794/2126 | NODE_12111 | 1794 | 1..1702 |
| tet(Q) | 90.41 | 1926/928 | 1028/2126 | NODE_13492 | 1647 | 721..1647 |
| tet(Q) | 99.88 | 1926/1702 | 1794/2126 | NODE_12112 | 1794 | 93..1794 |
| tet(X) | 99.58 | 1167/478 | 495/1367 | NODE_67268 | 495 | 18..495 |
| tet(X) | 100 | 1167/745 | 745/1367 | NODE_37914 | 745 | 1..745 |
| tet(X) | 98.02 | 1137/405 | 495/1367 | NODE_67267 | 495 | 91..495 |
| tet(40) | 99.67 | 1221/1223 | 1341/1421 | NODE_4242 | 3999 | 2430..3652 |
| tet(W) | 99.95 | 1920/1920 | 1991/2120 | NODE_3587 | 4535 | 2504..4422 |
| tet(W) | 94.80 | 1920/1904 | 1904/1920 | NODE_8607 | 2324 | 353..2256 |
| tet(O) | 99.69 | 1920/1920 | 1947/1947 | NODE_1035 | 11539 | 7962..9881 |
| tet(32) | 97.13 | 1920/1917 | 1341/1421 | NODE_4242 | 3999 | 92..2008 |
| aadS | 99.60 |  | 999/1064 | NODE_8334 | 2390 | N/A |
| dfrF § | 98.64 |  | 513/695 | NODE_8034 | 2460 | N/A |
| lnu(AN2) § | 99.72 |  | 710/713 | NODE_2939 | 5281 | N/A |
| mef(En2) § | 99.76 |  | 1245/1406 | NODE_2939 | 5281 | N/A |
| spw for ant9 § | 85.76 |  | 667/1010 | NODE_44 | 63779 | N/A |
| tet(44)* | 80.71 |  | 254/2123 | NODE_2781 | 5540 | N/A |
| vanS-D | 96.85 |  | 539/1146 | NODE_59322 | 539 | N/A |

**Sample HS25**

| Resistance gene | Identity % | Query/HSP | Alignment lenght / ref length (BLASTn) | Contig number | Contig length | Position in contig (from ResFinder) |
| --- | --- | --- | --- | --- | --- | --- |
| aph(3’)-IIIa* | 99.35 | 795/308 | 410/995 | NODE_25176 | 760 | 455..760 |
| lnu(C) | 98.99 | 495/496 | 677/675 | NODE_2614 | 3689 | 1198..1693 |
| erm(B) | 98.66 | 738/672 | 783/938 | NODE_132 | 24577 | 737..1406 |
| tet(W) | 99.38 | 1920/1920 | 2025/2120 | NODE_2346 | 3976 | 350..2269 |
| tet(O) | 99.43 | 1920/1920 | 1974/2030 | NODE_3797 | 2849 | 45..1962 |
| tetB(46) | 96.13 | 1737/646 | 747/1937 | NODE_25128 | 761 | 7..652 |
| dfrF § | 99.83 |  | 595/695 | NODE_6147 | 2034 | N/A |
| tet(32) | 80.61 |  | 495/2096 | NODE_2346 | 3976 | N/A |
| tet(Q) | 99.86 |  | 2126/2126 | NODE_579 | 9015 | N/A |

**Sample HS26**

| Resistance gene | Identity % | Query/HSP | Alignment lenght / ref length (BLASTn) | Contig number | Contig length | Position in contig (from ResFinder) |
| --- | --- | --- | --- | --- | --- | --- |
| ant(6)-Ia (aadE) | 99.81 | 867/518 | 545/994 | NODE_43679 | 868 | 1..517 |
| cfxA | 99.75 | 966/405 | 510/1166 | NODE_78172 | 597 | 1..404 |
| mef(A) | 95.07 | 1218/1218 | 1234/1427 | NODE_18986 | 1495 | 112..1329 |
| erm(B) | 100 | 738/738 | 938/938 | NODE_17594 | 1572 | 434..1171 |
| tet(32) | 100 | 1920/881 | 957/2096 | NODE_11193 | 2090 | 1210..2090 |
| tet(32) | 99.70 | 1920/996 | 1096/2096 | NODE_22901 | 1326 | 1..996 |
| tet(O/32/O) | 99.90 | 1920/1023 | 1039/2120 | NODE_6893 | 2836 | 1814..2836 |
| tet(O/32/O) | 99.50 | 1920/996 | 1095/2120 | NODE_1342 | 8380 | 7385..8380 |
| tet(Q) | 99.48 | 1926/1926 | 2126/2126 | NODE_2824 | 5056 | 2955..4880 |
| tet(40) | 99.75 | 1221/1221 | 1421/1421 | NODE_6893 | 2836 | 543..1763 |
| tet(W) | 99.95 | 1920/1920 | 2063/2120 | NODE_759 | 12500 | 822..2740 |
| aac(6’)-Ie / aph(2")-Ia § * | 99.35 |  | 460/1640 | NODE_17023 | 1603 | N/A |
| dfrF § | 100 |  | 659/695 | NODE_9718 | 2287 | N/A |
| lnu(AN2) § | 99.72 |  | 710/713 | NODE_862 | 11244 | N/A |
| mef(En2) § | 99.86 |  | 1406/1406 | NODE_862 | 11244 | N/A |
| spw for ant9 § | 85.43 |  | 597/1010 | NODE_12484 | 1949 | N/A |
| tet(O) | 96.62 |  | 1095/2120 | NODE_1342 | 8380 | N/A |

**Sample HS28**

| Resistance gene | Identity % | Query/HSP | Alignment lenght / ref length (BLASTn) | Contig number | Contig length | Position in contig (from ResFinder) |
| --- | --- | --- | --- | --- | --- | --- |
| aadA1* | 100 | 792/173 | N/A | NODE_48055 | 271 | 63..235 |
| catS | 97.46 | 492/118 | N/A | NODE_67036 | 262 | 145..262 |
| catS | 97.98 | 492/99 | N/A | NODE_67036 | 262 | 1..99 |
| erm(B) | 99.86 | 738/738 | 743/838 | NODE_2436 | 4415 | 1824..2561 |
| tet(40) | 99.59 | 1221/1211 | 1229/1421 | NODE_1461 | 6226 | 487..1697 |
| tet(44) | 98.37 | 1923/674 | 674/2123 | NODE_24355 | 672 | 1..672 |
| tet(C)* | 98.66 | 1191/672 | 672/1391 | NODE_24413 | 671 | 1..671 |
| tet(O) | 100 | 1920/1673 | 1773/2030 | NODE_2450 | 4397 | 2725..4397 |
| tet(Q) | 99.72 | 1926/1082 | 1082/2126 | NODE_14302 | 1082 | 1..1082 |
| tet(W) | 99.51 | 1920/1233 | 1333/2120 | NODE_3833 | 3181 | 1..1233 |
| tet(W) | 100 | 1920/786 | 886/2120 | NODE_8727 | 1651 | 866..1651 |
| tet(W) | 100 | 1920/404 | 404/2120 | NODE_27952 | 404 | 1..404 |
| cblA § | 99.54 |  | 653/1091 | NODE_23662 | 688 | N/A |
| mef(En2) § | 99.13 |  | 1034/1406 | NODE_14440 | 1071 | N/A |
| sul1 | 85.43 |  | 597/1010 | NODE_12484 | 1949 | N/A |
| tet(32) | 98.70 |  | 384/2120 | NODE_1461 | 6226 | N/A |
